# Evolutionary and Ecological Considerations on Nectar-Mediated Tripartite Interactions in Angiosperms and Their Relevance in the Mediterranean Basin

**DOI:** 10.3390/plants10030507

**Published:** 2021-03-09

**Authors:** Massimo Nepi, Daniele Calabrese, Massimo Guarnieri, Emanuele Giordano

**Affiliations:** Department of Life Sciences, University of Siena, Via P.A. Mattioli 4, 53100 Siena, Italy; daniel.calabrese@student.unisi.it (D.C.); massimo.guarnieri@unisi.it (M.G.); emanuele.giordano2@unisi.it (E.G.)

**Keywords:** nectar, Mediterranean plants, pollination, pollinators, bees, nectar-dwelling microorganisms

## Abstract

The Mediterranean basin hosts a high diversity of plants and bees, and it is considered one of the world’s biodiversity hotspots. Insect pollination, i.e., pollen transfer from male reproductive structures to conspecific female ones, was classically thought to be a mutualistic relationship that links these two groups of organisms, giving rise to an admirable and complex network of interactions. Although nectar is often involved in mediating these interactions, relatively little is known about modifications in its chemical traits during the evolution of plants. Here, we examine how the current sucrose-dominated floral nectar of most Mediterranean plants could have arisen in the course of evolution of angiosperms. The transition from hexose-rich to sucrose-rich nectar secretion was probably triggered by increasing temperature and aridity during the Cretaceous period, when most angiosperms were radiating. This transition may have opened new ecological niches for new groups of insects that were co-diversifying with angiosperms and for specific nectar-dwelling yeasts that originated later (i.e., Metschnikowiaceae). Our hypothesis embeds recent discoveries in nectar biology, such as the involvement of nectar microbiota and nectar secondary metabolites in shaping interactions with pollinators, and it suggests a complex, multifaceted ecological and evolutionary scenario that we are just beginning to discover.

## 1. Introduction

The Mediterranean basin hosts 25,000 angiosperm species and accounts for 7.8% of world plant diversity, while it covers only 1.6% of emerged land [1]. It is also considered a centre of bee speciation, hosting 3000–4000 species of bees [2,3]. The richness of flowering plants and pollinating insects results in particularly diverse plant–pollinator communities [4] in which wild bees are common pollinators for a large fraction of Mediterranean plants and are active almost all year round [4,5,6]. The main floral resources exploited by pollinators in the Mediterranean are pollen and nectar [3]. Although studies suggest that a high percentage of species reward pollinators only with pollen [7,8], nectar is in any case relatively common and a precious resource for many pollinators in dry Mediterranean habitats [9]. Regarding other floral traits, there is evidence of pollinator-driven selection of nectar attributes. In particular, nectar chemistry has been linked to specific preferences, in terms of sugar and amino acid profile, of pollinator guilds [10,11,12,13,14,15,16,17,18,19,20,21]. On the other hand, phylogeny and climate may impose constraints on nectar traits [9,22,23,24,25,26]. Thus, a number of factors of varying relative weight may shape nectar chemistry in different ecological and taxonomic contexts.

Here, we discuss nectar traits in angiosperms in the light of recent advances in nectar biology, ecology, and evolution, taking the Mediterranean plants as a case study. As far as the evolution of nectar is concerned, there is recent evidence that the plant–arthropod relationships involved in pollination and mediated by nectar-like secretions were established well before the rise of angiosperms [27,28]. Nectar evolved and diversified in angiosperms, allowing them to develop more efficient interactions with insects, and to override interactions already established previously by gymnosperms [28]. New and complex chemical profiles of floral nectar may have played a fundamental role in shaping such interactions. An outstanding concept of nectar biology was the demonstration that secondary compounds in nectar, which have been known since the 1970s and initially thought to be toxic deterrents of nectar thieves, may directly affect certain traits of forager behavior and therefore mediate plant–animal interactions [29,30]. A further complexity of nectar-mediated plant–animal interactions is the evidence that nectar-inhabiting microorganisms may be a third party in mutualistic relationships linking plants and nectar foragers. The ecological and evolutionary significance of this emerging tripartite relationship is still far from well understood [31].

All these things suggest a complex ecological and evolutionary scenario concerning floral nectar. It is evident today that in its interactions with organisms, nectar exhibits an array of potential functions far exceeding that of a simple food reward.

## 2. Nectar Sugars of Flowering Plants: An Evolutionary Hypothesis

Most plants of the Mediterranean basin flower in spring–summer and generally have nectar sugar profile high in sucrose [9,18,19,26,32,33]. It is said that high sucrose content in nectar can result from adaptation to Mediterranean spring–summer drought conditions, since high-hexose nectars consume more water than high-sucrose nectars per unit weight of sugar [9]. This consideration offers an opportunity to speculate about the evolution of this sugar profile that is shared by most of the angiosperms [34]. Sucrose-rich floral nectar was presumably not a plesiomorphic trait in early angiosperms, but it rather developed in response to climate changes in the Cretaceous period after the first appearance of angiosperms.

### 2.1. Rewards and Pollinators in the Mesozoic Era and in Early Angiosperms

Very early angiosperms were probably void of nectar. Fossil insect coprolites from the Early Cretaceous period, containing angiosperm pollen, suggest that the earliest (“ANITA” grade) angiosperms had dry stigmas and pollen was the only floral reward for pollinators [35,36,37]. Although controversies persist, a dry stigma is reported for *Amborella trichopoda*, the only extant species of Amborellales, which is considered to be the sister group to the rest of the flowering plants [36,38]. Some early diverging lineages (Annonaceae, Austrobaileyaceae, Chloranthaceae, Winteraceae) evolved stigma secretions independently [39]. In addition to providing an optimal medium for pollen germination, at least in some cases, these could function as rewards for pollinators, thus the name stigmatic nectar [37] or protonectar [39,40]. In the Early Cretaceous, flowers may also have produced “true” nectar, i.e., nectar produced by specialized complex secretory organs called nectaries [36]. In ANITA grade taxa, nectaries are relatively rare and scattered but diverse in structure (though relatively simple), pointing to the convergent evolution of nectaries in the basal angiosperm groups [35,36]. Nectaries became more common and their structure more defined and complex in clades that appeared soon after in the Early Cretaceous such as Magnoliales and Laurales [36]. In a 100 million-year-old flower assigned to Laurales, stamens or staminodes bear what are presumably paired nectariferous appendages [41]. Thus, the emerging picture of floral rewards in basal angiosperms highlights a variety of food that can be exploited by insects: pollen, stigma exudates (stigma nectar or protonectar), and nectar. The picture improves if we consider that insects fed on the pollination drop of gymnosperms, a sugary secretion produced by the ovule, well before the evolution of angiosperms. A certain consensus regarding a Mid Mesozoic (Early-Middle Jurassic) plant–insect pollination network was recently developed on the basis of new evidence from insect and plant fossils that highlights insects feeding on pollination drops of several extinct taxa of early seed plants [42,43,44,45,46]. This evidence suggests that insects fed on nectar-like secretions (pollination drops) about 40 million years before the rise of angiosperms.

In the Late Jurassic and Early Cretaceous periods, various beetles, early brachyceran flies, aglossatan mandibulate moths, sphecid wasps, and thrips consumed pollen grains and stigmatic or other sugary secretions, qualifying them as candidates for the earliest pollinators of angiosperms [35]. It is highly probable that beetle, moth, and fly pollination all evolved in angiosperms at much the same time [36]. The pollinator guilds that were active when the earliest angiosperms appeared, namely beetles, flies, thrips, and micropterigid moths, are still the main pollinators of living basal angiosperm families and of extant insect-pollinated gymnosperms [28,36]. Today, the hypothesis that the first flowering plants had a generalist pollination mode involving various insects as well as air currents is widely accepted [35,36,38]. Notably, all living and some extinct insect-pollinated gymnosperms are also, or are also thought to have been, pollinated by wind (ambophylous pollination) [28]. It is clear that the evolution of early rewards in angiosperms converged toward sugary secretions. The new condition of enveloped ovules that evolved in angiosperms led to the disappearance of sugary exudates (the pollination drop), and strong selective pressure may have promoted the provision of a new sugary exudate in flowers to cope with insects already adapted to feed on sugary surface fluids [27].

We do not know much about the chemistry of these early secretions. Direct evidence of the chemical profile of the pollination drop of insect-pollinated Mid Mesozoic gymnosperms is obviously impossible, but inference is possible. Mapping the compositions of ovular secretions of extant gymnosperms in a phylogenetic framework of seed plants led to the inference that early gymnosperms had ovular secretions that were a mosaic of those of modern species, with a high-fructose sugar profile [28].

Concerning stigma exudates, there is only one report on the sugar profile of a plant (*Uvaria macrophylla*) of an early divergent family (Annonaceae), revealing a hexose-rich (fructose-dominant, 72.2%) sugar profile with very little sucrose (8.4%) [47]. This sugar profile is in line with the beetle pollination that occurs in this plant, since the nectar of beetle-pollinated species is typically hexose-rich [48].

Interestingly, a hexose-rich sugar profile is common in the pollination drops of all extant ambophylous gymnosperms [28]. These taxa belong to the Cycadopsida and Gnetopsida, and the main pollinating insects are generally thrips, flies, and beetles [42,49,50]. In the Early and Middle Cretaceous period, Gnetopsida underwent a radiation that paralleled the diversifying angiosperms [42], and these taxa presumably competed to secure insect visits.

Therefore, gymnosperms (pollination drops) and early diverging angiosperms (stigma exudates) may have shared a hexose-dominant chemical profile of secretions that mirrored shared guilds of pollinators, such as Diptera, Mecoptera, Neuroptera, and Coleoptera [44].

Considering this scenario, it is also plausible that the secretions of the first “true” nectar-secreting angiosperms (i.e., angiosperm species in which the nectar is produced by a specialized organ, i.e., the nectary) had a hexose-rich sugar profile. In this regard, it is interesting to note that Diptera, which are the most common pollinators of today’s entomophilous Gnetophyta and also pollinate several angiosperm species due to their preference for hexose-rich nectar [22], experienced limited extinction in the interval when angiosperms became ecologically dominant [44]. Diptera probably shifted from earlier fluid-feeding on ovular secretions of gymnosperms to later nectar-feeding on angiosperms [50].

### 2.2. Evolution of Rewards along the Cretaceous

The further evolution of sugar-based secretions involved in rewarding pollinating insects was probably influenced by the nature of the secretion itself. The original functions of the ancient gymnosperm pollination drop were microgametophyte capture, delivery, germination, and ovule defense [51], all directly connected with plant reproduction. Subsequently, another function was acquired, i.e., insect rewarding, which can be considered a kind of exaptation for co-opting insects into feeding on these secretions [27]. However, chemical modifications to adapt to the new function were limited, since the maintenance of a suite of chemical and biochemical traits was necessary to accomplish the original functions (see also Section 3.1).

Something similar may be true for stigma exudates. The original function of the stigma was reception and interaction with the male microgametophyte. According to recent studies, the dry stigma is considered the basal condition in angiosperms [38]. Although involved in rewarding insects, stigma exudates still also needed to fulfill the original aim of receiving pollen and promoting its germination. Interestingly, stigma exudates of extant angiosperms are involved in a series of functions [47].

True nectar, i.e., a sugary secretion elaborated by a nectary, produced solely to interact with animals, without any direct connection to reproductive function, is a different question. It allows the nectar and nectary to readily adapt to new requirements for efficient interaction with the environment and animals. The nectary is independent of the ABC floral homeotic genes that are responsible for floral organ specification according to position [52]. During evolution, the “new” secreting organ is “free” to move about the flower in response to selection imposed by interactions with the environment and pollinators, and it was acquired and lost independently several times in the course of angiosperm phylogeny [39]. On the other hand, the nectar chemical profile can be adjusted to the requirements of specific pollinator guilds in different ways in phylogenetically related species, revealing high phenotypic plasticity [10,12,19,21]. Thus, nectar that became common in Late-Cretaceous flowers (see below) could be a more efficient tool to interact with pollinators than others employed by gymnosperms and early angiosperms in the Mid-Mesozoic and early Cretaceous, i.e., pollination drops and stigma exudates constrained physiologically by their direct involvement in the reproductive mechanism.

## 3. Evolution of Sucrose-Dominant Nectar Sugar Profile

If the floral nectar of early angiosperms (Early Cretaceous period) had a hexose-dominant sugar profile, and today, most angiosperms produce floral nectar in which sucrose is the dominant sugar [22,34], one may well ask what promoted the switch from hexose- to sucrose-rich floral nectar. There are arguably at least two drivers of the change in chemical profile. A first driver may have been climate changes in the Cretaceous period. It is widely accepted that this was a period of high global warmth, at least partly explained by elevated CO2 concentrations, which were estimated at eight to ten times current ones [35]. Associated with global warmth, arid or semiarid conditions were apparently widespread, particularly at low and middle-paleolatitudes and in the very first part of the Early Cretaceous and in the Late Cretaceous period [35]. Widespread aridity imposed constraints on plant–water balance, and angiosperms evolved traits that made them more drought-resistant [53]. One trait may have been an increase in sucrose in floral nectar (see Section 3.1).

A second driver may have been co-evolution with radiating angiosperms of new groups of pollinating insects, such as bees (Apidae s.l.), members of brachyceran flies (especially Bombyliidae, Empididae, and Syrphidae) and Lepidoptera [36]. These new groups of insects may have had different food preferences from older groups, i.e., sucrose rather than hexose. Angiosperm nectar may have evolved a new sugar profile to co-opt these new pollinators. On the other hand, the chemical shift in nectar sugar profile imposed by climatic changes may have opened new ecological niches that selected a new feeding ability of insects. This correlation was highlighted in a recent study analyzing nectar diversity and pollinators in extant taxa to determine how the nectar sugar profile evolved [54].

### 3.1. Climatic Changes in the Cretaceous Period and Effects on the Chemistry of Sugary Secretions and Flower Evolution

The chemical shift from the almost sucrose-free secretions of gymnosperms and early angiosperms to the sucrose-dominant nectar of later angiosperms was probably triggered by a rapid physiological adaptation to a warming trend from the Cretaceous period to about the Campanian age 83.6–72.1 Ma [35]. This trend is mirrored by the spread of aridity, especially at low and mid-paleolatitudes [35]. In turn, the new chemical–physical characteristics of the nectar may have brought an evolutionary advantage in interactions with pollinators.

On the other hand, gymnosperms were not prompt to adapt their pollination drops to the new conditions, due to the physiological constraints mentioned above (see Section 2.2). The original function of the gymnosperm pollination drop was essentially to capture airborne pollen and transport it into the ovule by reabsorbing the drop. Therefore, the maintenance of precise rheological characteristics is fundamental for a good probability of reproductive success in gymnosperms. The two fundamental physical characteristics in this process of pollen capture and drop resorption are viscosity and surface tension, which are both directly influenced by temperature. Viscosity is a measure of a fluid’s resistance to flow. It describes the internal friction of a moving fluid. Flow transport (such as resorption of the pollination drop) in plants is characterized by precise relationships between friction and inertial forces [55]. When pollen grains impact the surface of a pollination drop, they are submerged; then, they hydrate, swell, and sink to the micropyle, finally entering the ovule upon withdrawal of the drop [56]. All these process are optimized in an appropriate range of viscosities. Surface tension is a property of the surface of a liquid that allows it to resist an external force due to the cohesive nature of its molecules [57]. It determines the shape of liquid droplets. Maintaining a spherical shape is important for gymnosperm pollination drops, since it maximizes the area for capture of airborne pollen and thus the probability of pollination. High surface tension is also useful to establish an appropriate cohesion force to keep pollination drops on the micropyle in the absence of organs to hold them and notwithstanding strong air currents. Surface tension also affects the evaporation of liquids: evaporation usually depends on heat capacity, i.e., the thermal energy required to overcome inter-molecular attraction and other thermal properties [58]. Higher surface tension usually means that more energy is needed to overcome inter-molecular attraction, resulting in lower evaporation and conserving the area available for pollen capture. So for gymnosperms, maintaining low pollination drop viscosity and high surface tension is paramount for securing efficient pollen capture and transport into the ovule. The two traits are guaranteed by a pollination drop sugar profile generally showing a preponderance of monosaccharides and little or no sucrose [28]. For an equal solute weight per unit volume, glucose and fructose solutions are less viscous than sucrose solution [59]. The relationship between carbohydrates and the surface tension of their aqueous solutions is more controversial and less known. However, polymerized sugars have a lower surface tension than solutions of the corresponding monosaccharide, due to a lower possibility of forming hydrogen bonds, which are the main generating force of surface tension [60]. The surface tension of sugar solutions ranks as follows, in decreasing order: glucose, fructose, sucrose [61].

Due to an increasing trend of temperature (with sea surface temperature up to 35–38 °C) and aridity in the Cretaceous period [35,62,63], a hexose-dominant sugar profile may have posed water balance problems for plants. Since monosaccharide solution has a higher osmotic pressure than sucrose solution, the secretion of a given weight of solute consumes more water in the case of monosaccharides. The above functional constraints forced gymnosperms to maintain dilute pollination drops rich in monosaccharides and prevented them from responding to increasing aridity by evolving a sucrose-dominant secretion profile. However, angiosperm floral nectar did not have such constraints and could therefore evolve a sucrose-dominant sugar profile more suited to the changing climate. Nectar scents as olfactory cues for pollinators were another advantage of increasing the sucrose content of floral nectar [64]. Actually, the lower surface tension compared to glucose- and fructose-dominant solutions allowed a higher rate of release of volatile molecules. Angiosperms may also have increased the sugar content of their floral nectar, becoming much more attractive to pollinators while saving water and increasing their fitness under arid conditions. The most evident difference between gymnosperm pollination drops and angiosperm floral nectars concerns sugar concentration, which is generally much higher in the latter [28]. Of course, producing a secretion with a higher sugar content implies an extra cost. Angiosperms may invest up to 37% of the daily photosynthate in nectar production [65]. This extra cost may have consequences for reproduction, limiting the resources for plant growth and seed development [66]. Thus, flowering plants have to balance the tradeoff between nectar production and plant growth–reproduction.

It is noteworthy that in extant ambophylous gnetophytes, where insects contribute to pollination and the pollination drop acquired the function of rewarding pollinators, the total sugar concentration of the pollination drop is similar to that of angiosperm floral nectar. However, sucrose concentration is low in pollination drops of ambophylous gnetophytes, which is presumably due to constraints imposed by conservation of the original functions (capture and transport of airborne pollen) [35].

**Figure 1:**
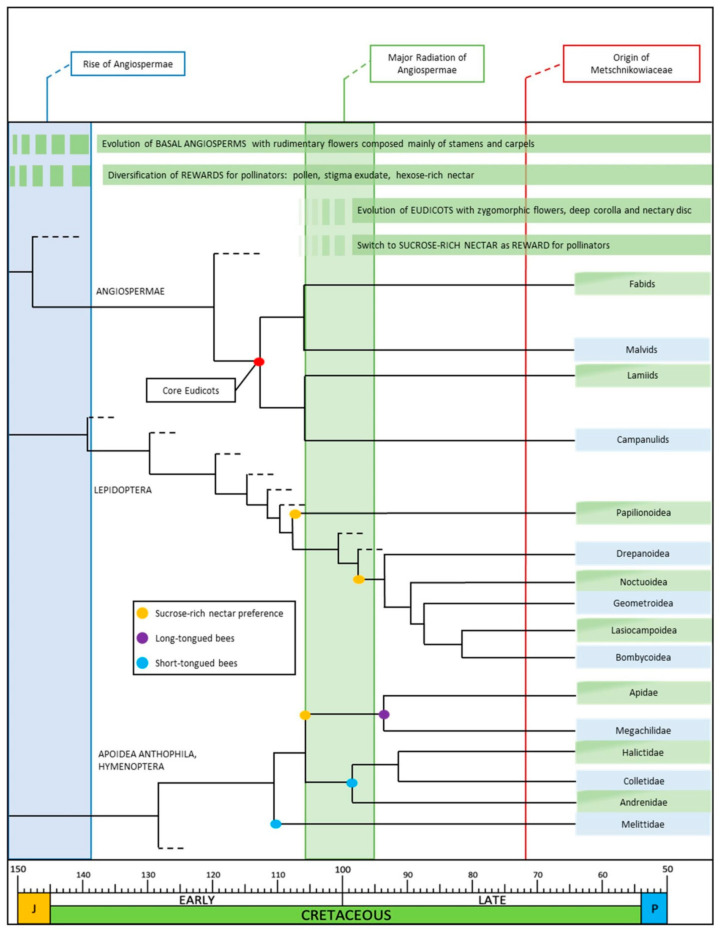
Annotated phylogeny of Angiosperms, Lepidoptera, and Hymenoptera summarizing the timing of evolution of principal traits involved in nectar-mediated flower–pollinator relationship. Dashed lines indicate non-eudicots clades or insect clades without sucrose-rich nectar preference. Phylogenetic data were obtained from [69,70,71].

The high-sucrose sugar profile of angiosperm floral nectar would result in a higher rate of evaporation in “open” flowers, such as those of the first angiosperms [42], where nectar, when present, was open to the air. As explained above for pollination drops, a higher rate of evaporation results in a quick change in nectar viscosity, which in turn affects the energy required by insects to feed on nectar, especially insects with a long proboscis [22]. Thus, better protection of sucrose-dominant nectar was probably a driver in the evolution of flower architecture, leading to floral nectar highly protected in deeper corollas and/or nectar reservoirs. Flowers also evolved the ability to fine-tune the concentration and hence viscosity of nectar by independently adjusting water and sugars by reabsorption [67]. From this perspective, the more elaborate sympetalous zygomorphic flowers and more efficient nectary positions, structures, and functions that evolved in the early Late Cretaceous [35,68] (Figure 1) can be considered adaptive traits to contain and protect a sugar solution rich in sucrose, while coping with more specialized pollinators. Such traits probably first evolved in insect-pollinated core eudicots in habitats with dry seasons similar to the present Mediterranean type. The first appearance in the fossil record of a receptacular nectary disc in a flower dates back 94–96 Ma (early Late Cretaceous, Cenomanian) and is common in extant and fossil flowers of core eudicots [36].

### 3.2. Evolution of New Groups of Insects and Their Foraging Behavior

According to the fossil record, the major ecological radiation of core eudicots took place in the early Late Cretaceous period, and by the Turonian–Coniacian age (93.9–86.3 Ma), they already dominated many plant fossil assemblages [35,68]. In the same period, important pollinator groups achieved significant diversity, and there was the first evidence of bees and glossate Lepidoptera [35,36]. As stated above, the new lineages of plants had floral traits better adapted to contain and protect a sucrose-dominant nectar. The evolution of corolla tubes imposed selection on the length of insect mouthparts to feed on concealed nectar. It is interesting that important insect families containing species with a long proboscis radiated around 75 Ma, when eudicots were already widespread. For example, Lepidoptera, for which nectar is the primary reward, appeared slightly before angiosperms, but most of them radiated in the Late Cretaceous [69,72] (Figure 1). Bees, for which the Mediterranean region is considered a diversity hot spot [2], are probably the group of pollinators that co-evolved most closely with angiosperms, and this relationship may account for much of the diversity of angiosperm flowers, as well as contributing to diversification of the major eudicot clades [35,73].

The first evidence of flowers with a long corolla tube, probably concealing sucrose-rich nectar, dates back to the Turonian, i.e., 89.8–93.9 Ma [54,74]. Today, such flowers and nectar are associated with anthophilous fauna, composed mainly of hummingbirds, moths, and long-tongued bees [22,54], which are not only able to access the nectar but are also efficient pollinators [75]. The very first long-tubed flowers were presumably exploited by animals with mouthparts sufficiently long to access concealed nectar.

Peters et al. [70] carried out an extensive study to investigate the phylogeny of Hymenoptera and estimated the time of divergence, in particular the events that marked key points of evolution. Apoidea Anthophila (bees) originated in the Early Cretaceous in a period spanning 124–111 Ma. Within the Anthophila, the first separation was between the Melittidae and the clade comprising all the other bees, which occurred around 111 Ma. Subsequently, around 106 Ma, there was the separation between the short-tongued bees (Halictidae, Colletidae, Andrenidae) and the long-tongued bees. Later (around 94 Ma), two long-tongued families, Megachilidae and Apidae, diverged, and both families underwent strong diversification. It is evident that in a period compatible with the existence of flowers with deep corollas of early eudicots, bees with long mouthparts were also present [76] and might have exploited concealed sucrose-rich nectar (Figure 1).

Various studies suggest that with fewer flower resources, the advantage of a long proboscis, expressed as a specialized phenotype, diminished or was lost, potentially driving the rapid evolution of bees toward a shorter proboscis [77]. On the contrary, the greater and diversified availability of floral morphologies, offered by radiation of the angiosperms, may have led to a greater degree of specialization in various apoid taxa, favoring the evolution of longer mouthparts. This would explain a possible shift involving pollinating insects, including bees that already collected pollen and nectar [78]. The host-plant shift that generated a large radiation event in Apoidea was also probably associated with a different digestive ability of the new groups of insects to exploit sucrose-rich nectar. In fact, it is recognized that changes in the form, structure, and chemistry of angiosperms with respect to more ancient groups of plants had an effect on the evolution of insect behavior, mouthparts, mode of digestion, and other features [35].

High invertase activity in the insect gut is required to fully digest and utilize sucrose as an energy source. Ancestrally, having little or no need to digest sucrose, bees may not have produced invertase or have had low gut invertase activity. Heil et al. [79] quantified the invertase activity of β-fructofuranosidase in ants of various species, obtaining results that underlined that the intensity of invertase activity is species-dependent and also depends on the sources from which the colony feeds. It may be concluded that invertase activity is genetically determined but adaptive, according to the food source, at least in ants. It is important to remember that ants (Hymenoptera: Formicidae) are phylogenetically very close to Apoidea s.l., and divergence of the two lineages occurred around 168 Ma [70]. In *Apis mellifera*, α-glucosidases, the major enzymes that exploit sucrose as substrate [80], may have adapted according to food source, such as ant β-fructofuranosidase, and they may have been boosted by the higher level of sucrose in the floral nectar of angiosperms.

It cannot be excluded that the ability to digest sucrose depends or is increased by the insect gut microbiota. In fact, it is known that the exploitation of certain food resources can be increased by symbiotic intestinal organisms [81]. There is a significant similarity of symbiotic intestinal bacteria in species of the Apinae subfamily [82], which is probably because they share a similar diet [82,83]. The host-plant shift caused by the appearance of core eudicots can be associated with bee speciation events [84], as well as with adaptation or the qualitative and quantitative modification of bees’ symbiotic microorganisms [85].

## 4. Nectar Secondary Compounds and Their Importance in Mediterranean-Type Ecosystems

Sugars are only one of the solutes in floral nectar: other known substances are amino acids, which are generally the most abundant after sugars, proteins, lipids, organic acids, and vitamins [86]. A major recent discovery in nectar biology has concerned a re-consideration of secondary metabolites (SMs) and their role in shaping plant–pollinator interactions [87]. Tannins, phenols, alkaloids, and terpenes have been detected in floral nectar in several angiosperm families since the 1970s and are considered to be toxic deterrents against predators, as well as a defense against microorganisms [31]. The concentrations of secondary metabolites in nectar are generally lower than in other plant parts, such as leaves, stems, or flowers, where they deter herbivores [88]. Plants that modulate concentrations of SMs in their tissues and secretions evolved strategies to deter herbivores (high concentrations of SMs) while attracting and manipulating mutualists (low concentrations of SMs) to maximize the benefits they obtained [29]. Two secondary metabolites are clear examples of such strategies, namely the alkaloids caffeine and nicotine. At high concentration, they are used by plants as deterrent molecules to keep away phytophagous insects [89]. The same alkaloids have been detected at low concentrations in the floral nectar of a few angiosperm species [90,91]. These concentrations in nectar have been found to have an important effect on insect neurobiology. In particular, they stimulate memory and associative learning of bees and bumblebees, thus potentially affecting their foraging behavior [90,91].

Non-protein amino acids γ-aminobutyric acid (GABA), β-alanine, and taurine were recently highlighted in floral nectar. These substances may directly affect insect nervous system activity and possibly insect behavior [30,92]. GABA is one of the main neurotransmitters in both vertebrates and insects [30]. GABA is a major neurotransmitter in vertebrates and insects [30]. Taurine and β-alanine are not neurotransmitters, but they may interact with receptor proteins on neurons [30].

GABA seems to have a special role in Mediterranean ecosystems. GABA is particularly common in the floral nectar of plants of the Mediterranean basin. Petanidou et al. [14] found GABA in the nectar of 63% of plant species of the phrygana community, which is a low dwarf shrubland plant association common in the eastern Mediterranean. GABA was detected in 82/82 species of the Lithospermeae tribe (Boraginaceae) having typical Mediterranean distribution at concentrations positively correlated with that of sucrose [93,94]. GABA seems to be related to specific pollinator guilds, such as long-tongued anthophorid, and andrenid bees, which are particularly diverse in the Mediterranean region, as well as anthomyiid and syrphid flies [14].

Several functions of GABA have been recognized in plants and animals. In plants, GABA is reported to be involved in response to pathogen attack and to accumulate after infection by fungi and bacteria [92,95]. Plants also use GABA to combat abiotic stress due to drought, salinity, low light, and low temperature [96]. Furthermore, GABA is an endogenous signaling molecule involved in the regulation of plant growth and development and in plant fertilization, where it drives pollen tube growth in pistil tissues [96,97].

In vertebrate and invertebrate animals, GABA is the most abundant inhibitory neurotransmitter [30]. Its inhibitory effect is at least partly due to binding of the molecule to ionotropic receptors, i.e., ion channels that change their permeability to ions after binding with the ligand. The flow of K^+^ or Cl^−^ along the channel hyperpolarizes the neuron, decreasing its probability of firing [30]. Interestingly, K^+^ is generally the most abundant nectar ion [20,98]. A diet containing GABA affects the survival and behavior of pollinating insects. Caged bumble bees, fed with artificial nectar containing GABA at concentrations similar to those occurring naturally in nectar, have a higher survival rate than control bumble bees [99]. In insects, GABA also limits excessive, potentially disruptive excitation states under conditions of stress [92,99].

As a result of its role in stress protection, GABA could have originated in plant tissues as an adaptation to increasing aridity in the Cretaceous period, similar to how sucrose dominates in floral nectar. As postulated for other secondary compounds in nectar, low concentrations of GABA may leak into nectar from the surrounding tissues as a pleiotropic effect, as postulated for other secondary compounds in nectar [92]. Pollinators, specifically bees, may impose a selection of specific concentrations of GABA if it has positive effects on their physiology, as suggested by Bogo et al. [99] and as observed for other nectar secondary compounds [90,91].

Nectars with appropriate concentrations and profiles of SMs presumably evolved and diversified in angiosperms and allowed them more efficient interactions with insects, overriding interactions already established in gymnosperms [28]. In this regard, it is interesting that ambophylous gymnosperms, which co-diversified with angiosperms, contain a non-protein amino acid (β-alanine) in their pollination drop, unlike wind-pollinated gymnosperms, the pollen drops of which are almost devoid of non-protein amino acids [28]. Testing the pollination drops of ambophylous gymnosperms for other secondary metabolites could shed light on the evolution of a “blend” of secondary metabolites in this group of plants, which competed with radiating angiosperms to secure pollinator visits. Although we know few nectar secondary compounds [30,87], discovering how they were shaped and how they influenced the evolution of plant–pollinator relationships could be an interesting topic for future research. A further development in this topic will be to assess the effect on insects of a mixture of secondary compounds, since the current studies are based on the provision of single molecules.

## 5. Nectar-Dwelling Microorganisms Contribute to Shaping Flower–Insect Relationships in Mediterranean Regions

For hundreds of years, pollination was considered a relationship between two partners; today, we know it involves other organisms besides plants and the animals they rely on for reproduction [100,101]. Microorganisms are an ‘‘unseen majority’’ in terms of numbers, species, and biomass in many ecosystems and may therefore play key roles in shaping community and ecosystem functions [102]. The chemical environment of nectar is a strong selective driver for the establishment of communities of microorganisms in nectar. It is assumed that a high concentration of nectar sugars, generating high osmotic pressure, is a strong limiting factor for the growth of microorganisms [103,104]. In addition to high sugar concentrations, the chemical environment of nectar is characterized by specific protein profiles, some with known antimicrobial or antifungal function [86], and by specific secondary metabolites (such as phenols, tannins, and alkaloids) with known antimicrobial activity [38]. Nonetheless, various microorganisms are able to grow in floral nectar, and some have selected floral nectar as their elective habitat, evidently overcoming these limiting factors. Nectar-dwelling microorganisms are generally a subset of those found on and in the pollinator body, and they contaminate the nectar while insects are foraging [44,104]. In any case, contamination by air vectoring or by contact with the corolla microbiota cannot be excluded [34]. The abundance and diversity of nectar-inhabiting microorganisms depends on pollinator activity and pollinator identity [104,105,106].

Yeasts are commonly found in plants in Mediterranean regions [107,108,109], and their frequency and abundance have been directly correlated with the proportion of floral visits by pollinators [103]. Bacteria also are frequent dwellers in floral nectar of Mediterranean plants and are generally associated with yeasts [110]. Specific yeast–bacteria interactions were recently recognized in nectar and may be responsible for a non-random assembly of nectar microbiota [111].

The presence of these microorganisms in floral nectar may have several consequences for plant–pollinator interactions by altering visual, olfactory, or gustatory cues [112]. Specifically these microbes can impact drastically the nectar chemistry. First, microorganisms may alter the sugar and amino acid profile of nectar as well as concentrations of secondary compounds [34,113]. Yeast metabolism causes a density-dependent decline in total sugar concentration of nectar, as well as changes in sugar composition. Fructose, glucose and sucrose concentrations may decline as microorganism density increases, but this effect depends on the microorganism identity and plant species [34]. In addition, effects on nectar chemistry are also influenced by the interaction between microorganisms. The growth rate of a specific microorganism, and thus its effect on nectar chemistry, may be strongly affected by the presence of other microorganisms [97]. Both primary and secondary compounds are known to influence pollinator foraging choices and behavior [31]. Through direct effects on the chemical profile of nectar, microorganisms may have indirect consequences for interactions between flowers and pollinators. Second, nectar-fermenting microorganisms produce a bouquet of volatiles that can be used by bumblebees and honey bee workers as a foraging cue [114,115]. Sugar catabolism by yeast populations dwelling in floral nectar can modify the thermal microenvironment in the flower, increasing internal temperature, favoring the dispersal of volatiles and thus increasing this chemical signal [115,116]. Increased emission may promote associative learning by pollinators of yeast-derived volatiles with floral rewards, thus making flowers more attractive [106]. In any case, volatiles produced by yeasts are species-specific, and their effects on pollinators may also vary [117]. On the plant side, it is not obvious that an increased flower visitation rate is mirrored by increased seed set or increased plant fitness [118]. Indeed, increased bumblebee visits to flowers of *Helleborus foetidus* inoculated with yeasts reduced fruit and seed set [114].

Yeasts of the genus *Metschnikowia*, particularly *M. gruesii* and *M. reukafii*, are common and abundant in floral nectar of Mediterranean plants pollinated mainly by bees [34,119,120]. Both yeast species produce volatiles that are highly attractive to honey bees and other insects compared to volatiles produced by other microorganisms [115,121]. These ascomycetous yeasts use nectar sucrose as a carbon source, reducing sucrose concentration and leaving unbalanced proportions of the monomers glucose and fructose [34,120]. Phylogenetic analysis dated the origin of the Metschnikowiaceae family to about 72 Ma (Late Cretaceous) [122], which is about 20–25 My after the major ecological radiation of eudicots. Although Metschnikowiaceae yeasts did not co-diversify with angiosperms, eudicots evolving sucrose-dominant nectar in the Late Cretaceous period may have created a new chemical environment that was later exploited by emerging Metschnikowia yeasts able to digest sucrose.

## 6. Concluding Remarks

Today, a nectar sugar profile rich in sucrose is a common trait of most angiosperms and a trait shared by most Mediterranean plants [22,26,34]. We postulate that this could have been an adaptation of angiosperm flowers to increasing aridity in the Cretaceous period, which also influenced the evolution of flowers with a corolla tube. The chemical shift from hexose-dominant to sucrose-dominant secretions gave angiosperms a chance to diversify their interactions with newly evolving groups of insects. Although a complex ecological scenario linking pollinating insects and gymnosperms already existed before the rise of flowering plants [41,42,43,44,45], the plasticity of the nectar chemistry of angiosperms gave them an advantage over gymnosperms, which needed to conserve the hexose dominance of their pollination drops. Thus, the idea that interactions with pollinators only regard angiosperms and are behind their evolutionary success is not completely true. In the Early Cretaceous, gymnosperms and angiosperms both interacted with pollinators, but the latter became more competitive, since they produce a more attractive reward for co-diversifying insects. The present high variability in floral traits has been interpreted as a result of plant adaptation to pollinator preferences [22]. Nonetheless, it has also been demonstrated that some pollinators, specifically bees, can learn and memorize associations between floral traits and the quality of rewards, thus overriding their innate preferences and changing their behavior according to what they encounter when foraging [123,124]. This foraging plasticity may have enabled pollinators to make the shift to the “new” floral resources that evolved in the Late Cretaceous period. The shift in nectar composition and the evolution of more elaborate flowers (bilateral symmetry and long corolla tubes) opened new ecological niches for emerging groups of insects but imposed a change in their digestive ability. To be exploited, the abundant sucrose in floral nectar required high invertase activity in the gut of nectar-feeding insects. Molecular studies into when genes involved in sucrose metabolism evolved in nectar-feeding insects, especially bees, would be of great interest and could strengthen our hypothesis. From the evolutionary point of view, it is interesting that in lepidopterans and coleopterans, genes encoding the sucrose-digesting enzyme β-fructofuranosidase were acquired from bacteria by horizontal gene transfer [125]. This suggests that the same may have occurred in long-tongued bees, enabling them to utilize sucrose-rich nectar. Studying the evolution of genes encoding for sucrose-clevage enzymes of bees could provide a support to our hypothesis.

Most probably, the switch from hexose-rich secretions to sucrose-rich nectar, evolved by core eudicots in the late Cretaceous, was the trigger for the establishment of interactions among Metschnikoviaceae yeasts, nectar, and foraging insects. Unraveling how the three partners influenced each other’s evolution since that time would be a valuable contribution to understanding the current diversity of insect pollination in angiosperms. A manipulative function of nectar was recently proposed: nectar containing neuro-active substances enables plants to manipulate animal behavior and increase the efficiency of the service they provide, and ultimately plant fitness [29]. With a third partner involved in the pollination scenario, it is reasonable to expect that microbes will also evolve to manipulate pollinators for their dispersal while driving floral evolution [100,126].
